# Roll-out of SARS-CoV-2 testing for healthcare workers at a large NHS Foundation Trust in the United Kingdom, March 2020

**DOI:** 10.2807/1560-7917.ES.2020.25.14.2000433

**Published:** 2020-04-09

**Authors:** Alexander J Keeley, Cariad Evans, Hayley Colton, Michael Ankcorn, Alison Cope, Amy State, Tracy Bennett, Prosenjit Giri, Thushan I de Silva, Mohammad Raza

**Affiliations:** 1 Sheffield Teaching Hospitals NHS Foundation Trust, Sheffield, United Kingdom; 2 The Department of Infection, Immunity and Cardiovascular Disease, The Medical School, The University of Sheffield, United Kingdom

**Keywords:** COVID-19, SARS-CoV-2, healthcare workers, diagnostic test

## Abstract

Healthcare workers (HCW) are potentially at increased risk of infection with coronavirus disease (COVID-19) and may transmit severe acute respiratory syndrome coronavirus 2 (SARS-CoV-2) to vulnerable patients. We present results from staff testing at Sheffield Teaching Hospitals NHS Foundation Trust, United Kingdom. Between 16 and 29 March 2020, 1,533 symptomatic HCW were tested, of whom 282 (18%) were positive for SARS-CoV-2. Testing HCW is a crucial strategy to optimise staffing levels during this outbreak.

The coronavirus disease (COVID-19) pandemic has posed an unprecedented challenge for healthcare systems throughout the world. As at 6 April 2020, Public Health England (PHE) have reported 51,608 cases in the United Kingdom (UK) and 5,373 deaths [1]. We report the results from the first fortnight following the roll-out of staff testing for COVID-19 at Sheffield Teaching Hospitals NHS Foundation Trust, UK.

## Establishing a programme for staff testing at Sheffield Teaching Hospitals NHS Foundation Trust 

Sheffield Teaching Hospitals NHS Foundation Trust (STH) is one of the UK’s largest foundation trusts, employing ca 17,000 individuals and providing a range of hospital and community services. On 17 March 2020, STH commenced testing of symptomatic staff for severe acute respiratory syndrome coronavirus 2 (SARS-CoV-2), coordinated by the Occupational Health department. Staff members presenting with an influenza-like illness (defined as a reported fever AND one of: cough, sore throat, runny nose, myalgia, headache) or persistent cough, were directed to self-swab in the on-site assessment pods previously used for testing of ambulatory patients in the community. Written and pictorial instructions were provided to staff to self-swab the back of the oropharynx and then insert the same swab 4–6 cm to the back of the nasopharynx. 

The Sigma-Virocult swabs (Medical Wire, Corsham, UK), containing viral transport medium, were transported to the hospital laboratory at least four times daily. Nucleic acid extraction was performed using MagNA Pure 96 (Roche Life Sciences, Basel, Switzerland). Real-time RT-PCR was performed using the ABI LightCycler (Applied Biosystems, Foster City, United States) with primers and probes specific for both RNA-dependent RNA polymerase (RdRP) and envelope (E) genes [2]. Samples with positive amplification curves for both RdRP and E, or samples with an E amplification curve alone and a cycle threshold less than 35, were considered positive. Samples with E amplification alone but a cycle threshold of 35 or greater were considered indeterminate and sent away for further testing. RNAaseP was used as an internal control to ensure good cellular content of the sample. 

The majority of test results were communicated to staff on the same or the next day. Staff with a negative SARS-CoV-2 test were able to return to work if they felt well enough to do so. The policy was authorised by hospital management on 17 March.

We present the data from the first fortnight of staff testing. Test results were obtained from electronic laboratory systems. Where available, details of staff roles and their work patterns since the onset of symptoms were obtained through their electronic clinical record. However, owing to the volume of work in the virology department, no electronic clinical records were created for positive staff from 22 March 2020. This report is a description of the standard clinical care provided to staff at Sheffield Teaching Hospitals. Following application of the NHS Health Research Authority algorithm (available at http://www.hra-decisiontools.org.uk/research/), it was decided that no formal ethical approval from the Institutional Review Board was required to produce this report.

## Roll-out of staff testing at Sheffield Teaching Hospitals NHS Foundation Trust

**Figure 1:**
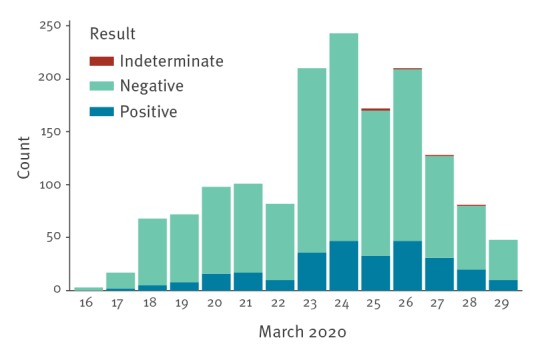
Daily breakdown of SARS-CoV-2 tests performed on staff at Sheffield Teaching Hospitals Foundation Trust, 16–29 March 2020 (n = 1,533)

In the week commencing 16 March, 451 staff members were tested for SARS-CoV-2 with 58 (13%) positive. In the following week, 1,082 additional staff were tested, and 20 staff were retested because of worsening symptoms, with 224 (20%) positive. Of the 20 staff retested, three were positive and 17 remained negative for SARS-CoV-2. Overall 282 of 1,533 (18%) staff tested positive, 1,246 of 1,533 (81%) tested negative and five of 1,533 (< 1%) had an indeterminate result. All samples contained sufficient human cellular material. Figure 1 details the daily breakdown of SARS-CoV-2 staff testing. 

## Analysis of the first week of staff testing (16 to 22 March 2020)

Among staff positive for SARS-CoV-2 for whom data were recorded (n = 52), 25 were nursing staff, eight were doctors, nine were other patient-facing clinical staff, nine were laboratory or secretarial staff and one worked in cleaning services. Regarding symptoms, where data were recorded (n = 48), 23 had not worked with any symptoms, seven had been at work when symptoms developed and had left work on the same day, and 18 had worked at least one full shift with symptoms which were subsequently attributed to COVID-19 disease. None of the staff who tested positive for SARS-CoV-2 required hospital admission at the time of their positive result. In the week when staff testing was started, between 16 and 20 March 2020, the Occupational Health department received 4,337 phone calls, compared with 998 calls between 9 and 13 March 2020.

## Contextualisation of staff testing at Sheffield Teaching Hospitals NHS foundation trust within UK policy and PHE guidance

**Figure 2:**
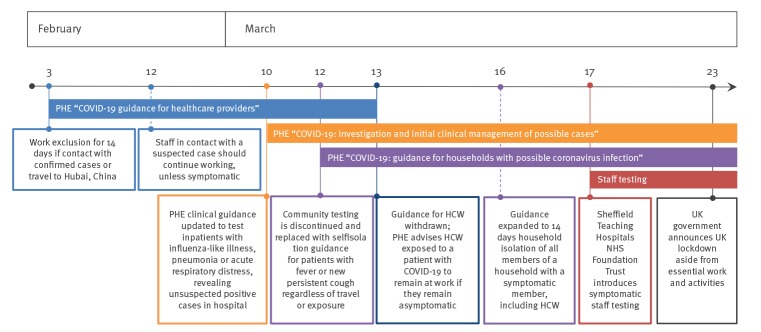
Timeline of key events relating to Public Health England COVID-19 guidance for healthcare workers, United Kingdom, March 2020

COVID-19: coronavirus disease; NHS: National Health Service; HCW: healthcare workers; PHE: Public Health England; UK: United Kingdom.

From 12 March 2020, in the UK, community testing was discontinued and any person with a fever or a new persistent cough was advised to self-isolate for 7 days, in order to enable UK laboratories to deliver priority testing for increasing numbers of hospitalised patients [3]. From 16 March, asymptomatic household members of any person with a fever or new cough were advised to self-isolate for 14 days [4]. The combined impact has meant that healthcare workers (HCW) with symptoms or with a symptomatic household member have been unable to work, exacerbating pre-existing staff shortages in frontline services in the National Health Service (NHS). In a survey of 5,194 doctors across the UK conducted over 24 h on 17 March, 12.8% were unable to work because they were symptomatic, and a further 15.1% were unable to work because of a symptomatic household contact (Michael Marks, personal communication, 26 March 2020). At that time, PHE guidance stated that frontline staff who had been exposed to patients with confirmed SARS-CoV-2 infection without wearing personal protective equipment should continue to work so long as they remain asymptomatic [5]. This scenario has led to anxiety among exposed staff. Furthermore, in these conditions, staff members with only mild symptoms may feel pressured to continue working. Figure 2 describes the timeline of key guidance impacting HCW in relation to the COVID-19 outbreak in the UK.

## Discussion

Our data demonstrate that the majority of staff who reported symptoms during the first fortnight of staff testing were negative for SARS-CoV-2. In a time where healthcare systems are particularly stretched, it is important to be able to maximise the available workforce. Facilitating testing may substantially decrease absence in staff when SARS-CoV-2 can be excluded. The sensitivity, and therefore the false-negative rate, of the two-gene target RT-PCR assay has not been formally described in the early stages of illness. However, SARS-2-CoV viral load in upper respiratory secretions is highest between 1 and 5 days from symptom onset, which is the optimal time to test symptomatic HCW [6]. If this test is negative, provided HCW are well and their symptoms have resolved (except for cough which is known to persist following any respiratory viral illness), it is reasonable that they return to work. Another major issue for healthcare staffing in the UK is that asymptomatic HCW must self-isolate for 14 days when they have a symptomatic family member. An additional boost to the workforce could be provided by access to testing for symptomatic household members of HCW. 

The nature of work performed by healthcare professionals means that they may be at increased risk of COVID-19 infection and can also transmit infection to vulnerable patients [7]. More than a third of staff had completed at least one shift while symptomatic. A limitation of this report is that exact symptoms have not been recorded. Therefore, precise assessment of proportion of staff meeting PHE criteria for self-isolation is not possible. However, during contact of staff members testing positive for SARS-CoV-2, we ascertained that most staff who had continued to work had only mild or non-specific symptoms at the time of clinical work. Early non-specific symptoms are challenging as HCWs may inadvertently expose other staff and patients before overt clinical symptoms manifest. Subclinical and asymptomatic infection has been reported, but its role in transmission is not clear [8-11]. Testing asymptomatic staff would have significant practical limitations and is not feasible with testing capacity in the UK. However, testing staff with mild or non-specific symptoms should be considered for infection control purposes, taking into account the practical implications of last-minute staff cover, optimal timing for swabbing to reduce false-negative results and laboratory capacity for testing [12]. Another limitation of this report is the lack of data on the roles of every staff member tested, in order to be able to provide a detailed breakdown of infection rate among different staff groups.

Testing of staff at our institution may also have provided a limited snapshot of SARS-CoV-2 prevalence in the community (i.e. those not requiring hospital admission) with mild influenza-like illness or persistent cough. While it is important that the provision of testing is carefully balanced with consideration of laboratory capacity, increasing laboratory capacity to allow widespread testing of NHS staff could be a vital tool in achieving adequate staffing during the COVID-19 outbreak, and reducing the risk of transmission to vulnerable patients.
